# Discrepant Results on Two Different Single Gene Non-invasive Prenatal Tests for Cystic Fibrosis: A Case Report

**DOI:** 10.1055/a-2844-4548

**Published:** 2026-04-20

**Authors:** James P. Hogg

**Affiliations:** 1 Department of Maternal-Fetal Medicine, Mississippi Perinatal, PLLC, Ridgeland, United States

**Keywords:** cystic fibrosis, non-invasive prenatal testing, prenatal diagnosis, fetal therapy, genetics, prenatal care, prenatal cell free DNA screening, carrier screening

## Abstract

**Background** Prenatal cell-free DNA (cfDNA) screening has revolutionized the prenatal detection of fetal aneuploidy and other conditions. Originally designed to identify risk for trisomy 21, cfDNA screening has expanded to other fetal aneuploidies, microdeletion syndromes, dominant de novo single-gene disorders, and now autosomal-recessive single-gene conditions. The advancement of cfDNA screening offers patients and providers more options for prenatal risk assessment.

**Case** A 34-year-old G2P1001 patient received a false-negative result from one single-gene noninvasive prenatal testing (sgNIPT), also known as prenatal single-gene cfDNA screening, for fetal cystic fibrosis, followed by a discrepant true-positive result on a different sgNIPT platform. The patient has a prior child affected with cystic fibrosis. The second laboratory returned a high-risk result for fetal cystic fibrosis, which was confirmed with diagnostic amniocentesis.

**Conclusion** Prenatal sgNIPT screening should be individualized after appropriate counseling on the benefits and limitations of available screening and diagnostic tests. While diagnostic amniocentesis remains the society-recommended gold standard for prenatal diagnosis of autosomal-recessive conditions, these new, noninvasive technologies provide more options for patients who either do not want to assume the risks associated with or to better guide decision-making for diagnostic testing.

## Introduction

With ever-increasing availability and rapidly expanding technology, prenatal cell-free DNA (cfDNA) screening has revolutionized prenatal detection of fetal aneuploidy since the first commercially available tests were introduced to the public in 2011. Since that time, cfDNA screening has expanded to include screening for microdeletions syndromes, de novo autosomal dominant single-gene disorders 1 2 and combination carrier screening and fetal evaluation for autosomal-recessive conditions including conditions such as cystic fibrosis, spinal muscular atrophy, and alpha and beta hemoglobinopathies. 3 4

CfDNA testing has expanded the ability to identify high-risk fetuses with noninvasive methods. While carrier screening of both partners and prenatal diagnostic testing (chorionic villus sampling [CVS] or amniocentesis) are the gold standard, recommended methods for screening and diagnosis, single-gene noninvasive prenatal testing (sgNIPT), also known as single-gene cfDNA screening, provides an option for patients and providers in certain clinical scenarios. When partner carrier screening cannot be completed (which has been reported to be the case in >50% of pregnancies in some studies) 5 6 7 or if the pregnant person or couple wants more information instead of or before undergoing diagnostic testing, sgNIPT screening can provide information and refine fetal risk.

However, these new technologies are still considered screening, not diagnostic, tests and patients and providers need to be aware of the uncertainty inherent in tests with limited validation data available. These tests are not currently standard of care and are not yet recommended by any obstetric or genetics professional societies. More evidence is needed to understand the performance and clinical utility of prenatal sgNIPT screening for recessive conditions. Here, we present a case of a patient who received discrepant sgNIPT screening results from two separate commercial laboratories, followed by confirmatory diagnostic testing.

## Case

We present a case of a 34-year-old gravida 2 para 1001 who presented for maternal–fetal medicine (MFM) consultation and level II ultrasound at 19 weeks secondary to history of prior pregnancy affected by fetal/neonatal cystic fibrosis. The patient and her partner were both found to be carriers of cystic fibrosis during her first pregnancy after concern for fetal echogenic bowel prompted referral to MFM. She underwent diagnostic amniocentesis that confirmed fetal cystic fibrosis. Her child is now 2 years old and doing well on elexacaftor/tezacaftor/ivacaftor (Trikafta). Due to her child’s diagnosis of cystic fibrosis, she saw reproductive endocrinology and infertility and was planning to undergo in vitro fertilization with preimplantation genetic testing for monogenetic disorders for her next pregnancy. Prior to egg retrieval, she spontaneously conceived.

She underwent Unity Complete Fetal Risk screening (BillionToOne, Inc., Menlo Park, CA) at her primary obstetrician’s office at 11 weeks’ gestation. The technology uses cfDNA to determine fetal risk of aneuploidy and a select few autosomal-recessive conditions including cystic fibrosis. Her sgNIPT testing returned “decreased risk” for fetal cystic fibrosis, with a reported risk of 1/51 and fetal fraction of 6.5%.

At the time the test was sent, the Unity test was the only sgNIPT screening available on the market. Shortly after she received her results, a new sgNIPT test for autosomal-recessive conditions was released (Fetal Focus, Natera, Inc., Austin, TX). The patient and her partner had previously undergone carrier screening with Horizon Carrier Screen (Natera, Inc.) during her first pregnancy and were both determined to be carriers of the common *CFTR* F508del variant. At her MFM appointment at 19 weeks’ gestation, she was counseled on the option of amniocentesis as well as the option of sending the novel Fetal Focus test. Sending the novel test was the patient’s request and was not paid for by the testing laboratory. A sample was sent for testing at 19 weeks’ gestation.

Fetal Focus returned a high-risk result for fetal cystic fibrosis with a reported risk of 92/100. The fetal fraction was 13.0% and the predicted fetal genotype was homozygous F508del. After further counseling, she desired to proceed with diagnostic amniocentesis with targeted variant analysis, which confirmed the diagnosis of fetal cystic fibrosis with homozygous F508del at 23 weeks’ gestation. She was followed closely with serial fetal growth ultrasounds and fetal testing. Approval for prenatal CFTR modulator treatment was pursued and successfully obtained in the third trimester and she began treatment around 32 weeks.

## Discussion

SgNIPT screening for autosomal-recessive conditions holds the potential to provide fetal risk information to pregnant patients who cannot or do not want to pursue traditional carrier screening and diagnostic testing. However, as this case illustrates, false-negative results are possible, and the performance of these tests are not yet well characterized.

The technology underlying one of the commercially available tests (BillionToOne, Inc.) relies on the molecular detection of known common variants and has a stated limitation in detecting rare homozygous variants in *CFTR* . 3 4 8 However, this case is of a reproductive couple where both partners were known carriers of *CFTR* F508del. This common variant accounts for >60% of *CFTR* variants in the U.S. population. 9 Therefore, detection of at-risk fetuses with this variant is crucial for the utility of this test. However, there have been three additional false-negative cases reported in the literature, 10 all of which were determined through newborn screening to be homozygous or heterozygous for F508del.

Professional society guidelines do not yet recommend prenatal sgNIPT screening, due to the lack of evidence supporting the clinical performance of these tests. 11 The current published studies of Unity (BillionToOne, Inc.) were retrospective 3 4 12 and only collected outcomes on a small minority of the tested cohort (1 and 13%) in an enriched population. 13 Multiple different types of outcomes were used beyond molecular genetic testing, including newborn screening, physical exam, and provider or patient self-report, further reducing the validity of these studies. In order to support responsible future adoption of this technology, prospectively designed studies with molecular genetic testing outcomes on all participants are needed.

Despite these limitations, sgNIPT screening for recessive conditions still holds promise in certain clinical scenarios. Carrier screening for autosomal-recessive conditions has well-established clinical utility, 14 15 16 especially when performed preconception. However, in reality, a vast majority of carrier screening occurs during pregnancy, 6 7 17 and more than half of partners of pregnant carriers do not complete screening due to barriers such as lack of partner insurance coverage, availability, or knowledge of pregnancy. 5 6 18 19 Additionally, known carrier–carrier couples may seek additional information before or instead of diagnostic testing. In the case presented here, the risk refinement information provided by the second sgNIPT screening results helped the pregnant patient in her decision to ultimately pursue amniocentesis. Conversely, the false reassurance from the first sgNIPT may have deterred her from pursuing CVS at an earlier gestational age. Patient hesitancy to pursue diagnostic testing is not uncommon 7 20 and highlights an unmet clinical need that sgNIPT has the promise to fill.

Timely prenatal diagnosis of some autosomal-recessive conditions, including cystic fibrosis, has implications for prenatal management. Frequent ultrasound monitoring for meconium ileus is recommended and preparation can be made for neonatal assessment and care. Additionally, there are emerging investigational prenatal therapies with promising results from off-label use of the CFTR modulator elexacaftor/tezacaftor/ivacaftor (Trikafta), with case studies showing the reversion of meconium ileus and preservation of pancreatic function in some affected neonates. 21 In this case, early detection and diagnosis were crucial in the patient obtaining approval for in utero treatment.

As the technologies underlying sgNIPT screening continue to improve, test performance is clarified, and prenatal therapies emerge, sgNIPT screening has the potential to make a profound impact through earlier detection and improved outcomes for neonates affected by autosomal-recessive conditions.
